# Interpretation of microarray data in cancer

**DOI:** 10.1038/sj.bjc.6603673

**Published:** 2007-03-06

**Authors:** S Michiels, S Koscielny, C Hill

**Affiliations:** 1 Department of Clinical and Translational Research, Biostatistics and Epidemiology Unit, Institut Gustave Roussy, Villejuif, France

**Keywords:** microarray, validation, prognostic, predictive

## Abstract

Microarray studies aim at identifying homogeneous subtypes of cancer patients, searching for differentially expressed genes in tumours with different characteristics, or predicting the prognosis of patients. Using breast cancer as an example, we discuss the hypotheses underlying these studies, their power, and the validity and the clinical usefulness of the findings.

Microarrays have been described as a technology that will revolutionise medicine with the ultimate goal to develop effective treatments or cures for every human disease by 2050 (Ioannidis, 2005a). It has also been suggested that they could allow the testing of new drugs in clinical trials including only a small number of patients (Liu and Karuturi, 2004).

The main objectives of microarray studies are (1) to identify homogeneous subtypes of a disease on the basis of gene expression, or (2) to find genes that are differentially expressed in tumours with different characteristics or (3) to develop a rule on the basis of gene expression allowing the prediction of patient prognosis or of the response to a particular treatment.

Using pioneering work on breast cancer as an example, we shall review some of the problems in interpreting the results of these three types of study and discuss the validity, the possibility of overinterpretation, and the clinical usefulness of the findings.

## OBJECTIVES OF MICROARRAY STUDIES

### Identification of homogeneous subtypes of cancer

On the basis of microarray data, breast cancers have been divided into several subgroups using cluster analysis (Perou *et al*, 2000). A commonly used hierarchical clustering method starts by defining a distance between two breast tumours as a function of the difference in gene expression. One then regroups the two closest tumours and proceeds by regrouping tumours to obtain a cluster tree, which can be split into branches by selecting a cutoff distance. There are many algorithms available for clusterisation, and for a given algorithm there are many ways to define a cutoff distance. Furthermore, even in the case of random noise, the technique produces a cluster tree (Miller *et al*, 2002). It is thus very difficult to know if what is observed is a characteristic of the sample considered or would be reproducible in another similar collection of tumours. Interpretation of such studies is an open-field and experts agree that clusterisation is overused in the microarray field (Allison *et al*, 2006).

The clustering of breast cancer tumours has identified the three following main groups: oestrogen-receptor positive tumours (luminal), oestrogen-receptor negative and Her2-positive tumours, and oestrogen-receptor negative and Her2-negative tumours. It has been proposed to subdivide these main groups into more subtypes. The main groups happen to correspond to a well-known clinical classification, but there is complete circularity in the argument: one clusters tumours on the basis of gene expressions and then ‘validates’ the clusterisation by superimposing known classification. Some have considered that the clinical confirmation of the main groups was sufficient to accept the hypothesis that further subdivision will also lead to clinically meaningful classifications. The interpretation of what is clinically meaningful remains to be specified. An approach that exploits those clinical characteristics from the beginning will be more efficient if one is trying to identify groups of patients with homogeneous prognosis or groups of patients who will benefit from a given treatment.

### Finding genes that are differentially expressed in tumours with different characteristics

The principle is to find the genes that are most differentially expressed between two (or more) classes of tumours with different characteristics: for instance, between tumours from 34 breast cancer patients who developed a distant metastasis within 5 years after surgery and tumours from 44 patients who did not (van't Veer *et al*, 2002). A statistic measuring the difference in gene expression between the two types of tumours is selected. Genes are then ranked according to this statistic, starting with the most differentially expressed gene. A cutoff is selected leading to a list of genes most differentially expressed. Van't Veer selected the 70 genes with the highest correlation with a distant metastasis status at 5 years.

#### Many false positive genes

When one applies a statistical test for each gene, the number of tests performed is equal to the number of genes. If 10 000 genes are studied and none are really associated with the characteristics under study, then, taking the usual 5% limit for a significant *P*-value, one expects 5% of the genes, that is, 500 genes to appear as significantly associated with the characteristics, all being false positives. One solution to reduce the risk of false positives is to select more stringent rules to define statistical significance. For instance, Benjamini and Hochberg (1995) suggest to rank the genes according to the *P*-values, starting with the most significant, and to compare the *i*th *P*-value *p*_*i*_ to 5% × *i*/*n*, where *i* is the rank in the list and *n* is the total number of genes. Under some hypotheses, this limits to 5% on average the proportion of false positives among the genes declared significant, that is, the false discovery rate (FDR) is 5%. The FDR in a microarray study comparing two groups depends on (1) the proportion of truly differentially expressed genes, (2) the distribution of the true differences, (3) the variability of the gene expression and (4) the sample size.

#### Sample size

The sample size is the only parameter of the design of a study that is under the experimenter's control. Pawitan *et al* (2005) studied the theoretical relation between the FDR and the sample size in a realistic situation with 200 genes truly differentially expressed between two groups (twofold change in expression) among 20 000 genes. They selected the 200 genes most differentially expressed between the two groups. With five patients per group, they obtained an FDR of 91%, which means that 182 of the 200 genes selected were false positives. If one wants to reduce the proportion of false positive to the usual 5% level, one needs 56 patients per group. When the number of truly differentially expressed genes is smaller or when the fold changes are smaller, a larger sample size is needed.

#### Instability of gene lists

We reanalysed the data from the study by van't Veer *et al* (2002) by drawing repeatedly at random a sample of 78 patients out of the total population of 97 patients (Michiels *et al*, 2005). For each sample, we calculated a ‘gene signature’ defined as the 50 genes most correlated with the prognosis (Box 1). We repeated this procedure 500 times and counted how many times a gene was part of those 500 signatures. Among the 70 genes from the published signature, 14 were included in more than half of the 500 replications, 10 genes not in the published signature were also in more than half of the replications. Furthermore, 564 different genes were included in at least one signature. Thus, the molecular signature is not unique and strongly depends on the selection of patients. We observed that every set of patients led to a different list of genes in the signature. The reason is that there are many genes with more or less the same correlation with the outcome; therefore, the list of the most correlated genes changes drastically when a different patient set is used.

#### Validation by RT–PCR of list of genes identified by microarray

DNA microarrays are not the only available technique for identifying genes with different levels of expression in tumours with different characteristics. For instance, quantitative reverse transcriptase PCR is considered as a reference method to measure the mRNA expression of genes. Many authors including us (Koscielny *et al*, 2005) have verified that the expression of genes measured by microarrays do correlate with the expression of the same genes measured by quantitative RT–PCR. However, this is the least one can expect, otherwise one of the two measurement techniques would be unreliable. Selecting from microarray data the most differentially expressed genes between tumours with different characteristics, and then re-measuring the expression of these genes on the same tumours by RT–PCR does not validate the list of genes as associated with the specific tumour characteristic. This pseudovalidation has been described by Allison *et al* (2006) ‘as a highly questionable practice that stems more from tradition than careful thought’.

### Development of a prediction rule based on gene expression

The aim of this type of study is to find an equation combining the expression of a number of genes, to predict a clinical outcome. In van't Veer's study, a prediction rule for prognosis based on the expression of the 70 genes was determined from data on 78 node negative breast cancer patients and then evaluated on another 19 patients.

#### Choice of prediction rule

Many complicated prediction rules have been suggested in the microarray literature. The result has been adequately described by Allison *et al* (2006) as a statistical tower of Babel. For the time being, we consider that the priority is to understand the limitations of the methods in use, rather than to develop complex statistical methods.

Some microarray analysis packages present systematically the results of several classification methods for a single data set. It is then very tempting to publish only the best looking result, leading to a biased evaluation of the performance of the prediction rule (Ioannidis, 2005b). In principle, there is no biological or mathematical reason why one particular classification method should be better than another for the prediction of the outcome of cancer patients based on microarray data and there are many possible solutions in the multidimensional gene expression space.

To avoid that pitfall the classification method used should be selected a priori, and defined in the protocol (McShane *et al*, 2005). The description of a classification method should include the method used to define the number of genes to be selected as well as the type of equation used to combine their expressions.

#### Evaluation of the performance of the classification rule

Having defined a prediction rule, the next step is to evaluate its performance; and this is most often evaluated by the proportion of misclassified patients. If this evaluation is conducted on the very data used to define the rule, one gets overoptimistic results, as the rule is optimised for this particular sample called the training set. The solution is to study an independent sample called the test set.

To avoid having to find an independent sample, a common practice is to split the original sample. This can be done once or several times using a resampling technique. The most popular resampling technique is the leave-one-out cross-validation method (Simon, 2003), but one could also leave-many-out and do this repeatedly (Michiels *et al*, 2005).

Each time patients are left out, the entire procedure of selecting the genes and constructing the prediction rule has to be repeated from the beginning, otherwise the proportion of misclassified patients would be underestimated (Simon, 2003). Consequently, the prediction rule is different each time and therefore not the same as the prediction rule developed on the entire sample whose performance one actually wants to evaluate. The instability is even worse in small sample settings (Braga-Neto and Dougherty, 2004). Thus, splitting the original sample in many ways is a first step in the right direction, but is not an independent validation, which is the only way to evaluate the performances of the prediction rule developed from the entire sample.

#### Replication in an independent population

Providing evidence that a prediction rule works satisfactorily on patients other than those used to define the rule is an external validation (Altman and Royston, 2000). Some basic scientific rules need to be applied:

- The inclusion criteria must be the same as in the study defining the prediction. (After a first validation using the same criteria as in the original study, it may be interesting to test the prediction rule in a population defined with broader criteria, to study the validity of the results in an extended population)
- The clinical end point must be the same.
- The prediction rule used to classify must be the rule defined in the initial study and it should be described in the protocol of the validation study. The description includes
  - the list of genes selected,
  - the method used to measure their expression,
  - the equation and
  - the cutoff used to classify a new patient in the high-*vs* low-risk group

The 70-gene signature in van't Veer *et al* (2002) study predicting the metastasis status 5 years after the diagnosis in node-negative breast cancers has been validated several times.

Van de Vijver *et al* (2002) studied a consecutive series of 295 patients, including both node-positive patients and node-negative patients, whereas 61 out of the 151 node-negative patients were already in the first study (Ransohoff, 2004). The clinical end point was slightly different since in the first study all patients had been followed-up for 5 years, which was not the case in van de Vijver's study. The prediction rule was almost the same but the cutoff values were different for the 61 patients in the original study and for the other patients. If one evaluates the performance of the prediction rule using only the 180 patients who (a) were not in the original study and (b) had a known metastasis status at 5 years, one obtains a sensitivity, or probability that a patient who will relapse is classified as high-risk, of 93% (95% CI: 81–99%) which is good and a specificity, or probability that a patient who will not relapse is classified as low risk, of 53% (44–61%) which is poor.

Recently Buyse *et al* (2006) confirmed these results on 307 node-negative breast cancer patients with a good sensitivity of 90% (78–95%) and a poor specificity of 42% (36–48%).

Another validation was conducted on 96 patients violating all rules: measuring gene expression by RT–PCR instead of microarray, using 60 genes instead of 70, a different equation and a different cutoff (Espinosa *et al*, 2005).

Paik *et al* (2004) developed a 21-gene prediction rule for the prognosis of node-negative, oestrogen receptor-positive breast cancer patients treated with the hormonal treatment tamoxifen. A large training set was used that included patients from the tamoxifen-only arm of the NSABP-20 trial comparing tamoxifen to tamoxifen plus chemotherapy. The prognostic value of this rule was confirmed on a population of patients from the tamoxifen arm of another NSABP trial (B-14). Recently, Paik *et al* (2006) attempted to show that this rule could also predict the benefit from chemotherapy. They used data from the two arms of the NSABP-20 trial, observed that the prediction rule was a better predictor of recurrence-free survival in the tamoxifen-only arm as compared to the tamoxifen plus chemotherapy arm, and interpreted this result as a demonstration that the rule ‘predicts the magnitude of chemotherapy benefit'. However, a more obvious interpretation is that a prediction is optimal for the patients in the training set used for its construction (Ioannidis, 2006).

#### Clinical use of the results

At the present time, the prognosis of node-negative breast cancer is known to depend on the age of the patient, on the size of the tumour, its pathological grade and the presence or absence of hormonal receptors in the tumour. It is important to verify that the gene signature adds to these prognostic factors (Simon, 2005). Many of the different published gene signatures predicting distant-metastasis free survival in breast cancer (van't Veer *et al*, 2002; Paik *et al*, 2004) have been found to be significantly correlated with tumour grade (Fan *et al*, 2006). One must therefore study whether these signatures add prognostic information to the grade. It is not sufficient to perform a multivariate regression analysis, for instance a Cox's regression, comparing the effects of the clinical prognostic factors and of the signature (as in van de Vijver *et al*, 2002 or in Wang *et al*, 2005), and to show that the gene signature is ‘more significant’ than the clinical factors in this model. What is required is to compare the predictive accuracy of the two multivariate models with and without the gene signature. It has been shown on the 234 patients from van de Vijver *et al* (2002) who were not in the first study that the gene signature added a moderate but not significant improvement in predictive accuracy when added to the prognostic factors: age, nodal involvement, oestrogen receptor status and tumour grade (Dunkler *et al*, 2007).

## CONCLUSION

The search for molecular gene signatures is based on the assumption that a clear distinction between tumours that will relapse and those that will not is possible using gene expression. The results of one of the first prognostic microarray studies in breast cancer (van't Veer *et al*, 2002) were considered as proof of this concept. Indeed, some authors thought that gene expression profiles would allow one to predict, with 90% accuracy, whether the tumour will remain localised or whether the patient will experience metastases and disease relapse (Bernards and Weinberg, 2002) and that the metastatic capacity of breast tumours is an inherent feature of the primary tumour (van't Veer and Weigelt, 2003).

Unfortunately, the actual performance of prediction rules using gene expressions is not as good as initially published, and the lists of genes are highly unstable (Michiels *et al*, 2005). So far, most prediction rules using gene expression have not provided a substantially and significantly improved prognostic classification when compared to conventional prognostic factors (Eden *et al*, 2004; Dunkler *et al*, 2007). These results could be interpreted as disproving the initial assumption.

In conclusion, we appreciate the efforts of the above-cited pioneering work in breast cancer. However the prognostic value of the gene signatures identified seems to have been oversold, maybe because of the enormous investments and because of the high expectations in a new technology. The results are correct in so far as the published combinations of genes do have some prognostic value. However, many other gene combinations would be as good and so far none have been shown to add much to the clinical information that is routinely available. The example of breast cancer illustrates a problem that is central to the interpretation of microarray data. The hypothesis underlying each study should be stated clearly and the primary objective of a study should aim at its rejection. Studies with a solid experimental design and larger sample sizes are required before gene expression profiling can be used in the clinic to predict outcome.

## Figures and Tables

**Box 1:** A critical view of microarray vocabulary

*Prognostic marker:* a biological marker which is associated with a specific outcome, for instance a gene which is overexpressed (marker positive values) in patients who will develop metastases and not in patients remaining free of metastasis (marker negative values). The measurement of the expression of this gene allows the prediction of the risk of metastasis.

*Predictive marker:* expression used either to designate a prognostic marker, or to designate a marker predicting the usefulness of a given treatment. In that second case, the benefit of the treatment is greater for the patients say with positive marker values, or even restricted to these patients. To establish this result, the changes in the treatment effect with the marker values must be studied in the setting of a controlled clinical trial in order to compare the benefit of the treatment with positive or negative marker values. If one wants to select a treatment for a group of patients on the basis of gene expression markers, these must have been demonstrated predictive of the effect of this treatment.

*Individualised treatment:* is considered as the ultimate goal of microarray studies. The hypothesis that cancer treatment can be individualised cannot be tested. If one assumes that patients vary truly randomly in their response to a drug, individual response cannot be predicted (Senn, 2004).

*Signature:* searching for ‘the signature’ predicting the risk of distant metastasis within 5 years after diagnosis implies that there is a unique molecular fingerprint for this risk. This is an extremely strong assumption.

*Validation:* a study designed to confirm the results of a previous study, in order to reduce the play of chance and the potential for biases (Ransohoff, 2004, 2005). Common mistakes with validation studies have been:
• To include part of the initial sample of patients in the validation study
• To include other type of patients in the validation study than in the initial sample
• To use another measurement technique (rt–PCR *vs* microarray)
• To change the prediction rule by adapting it to the new sample of patients through changing the list of genes, or the equation, or the cutoff
